# Late Outcomes of Undiagnosed Unilateral Condylar Hyperplasia and Reoccurrence of Mandibular Asymmetry

**DOI:** 10.3390/diagnostics14101014

**Published:** 2024-05-15

**Authors:** Kamil Nelke, Wojciech Pawlak, Klaudiusz Łuczak, Maciej Janeczek, Edyta Pasicka, Jan Nienartowicz, Grzegorz Gogolewski, Maciej Dobrzyński

**Affiliations:** 1 Nelke Privat Practice of Maxillo-Facial Surgery and Maxillo-Facial Surgery Ward, EMC Hospital, Pilczycka 144, 54-144 Wrocław, Poland; wojciech.pawlak.mfs@gmail.com (W.P.); klaudiusz.luczak@gmail.com (K.Ł.); 2 Academy of Applied Sciences Angelus Silesius in Wałbrzych, Health Department, Zamkowa 4, 58-300 Wałbrzych, Poland; 3 Department of Biostructure and Animal Physiology, Wrocław University of Environmental and Life Sciences, Kożuchowska 1, 51-631 Wrocław, Poland; maciej.janeczek@upwr.edu.pl (M.J.); edyta.pasicka@upwr.edu.pl (E.P.); 4 Private Practise of Maxillo-Facial Surgery, Romualda Mielczarskiego 1, 51-663 Wrocław, Poland; nienartowicz@gmail.com; 5 Department of Emergency Medicine, Wrocław Medical University, Borowska 213, 50-556 Wrocław, Poland; grzegorz.gogolewski@umw.edu.pl; 6 Department of Pediatric Dentistry and Preclinical Dentistry, Wrocław Medical University, Krakowska 26, 50-425 Wrocław, Poland; maciej.dobrzynski@umw.edu.pl

**Keywords:** asymmetry, mandible, condylar hyperplasia, SPECT, CBCT

## Abstract

Unilateral condylar hyperplasia (UCH) is a rare cause of asymmetrical mandibular overgrowth because of the presence of an atypical growth in the affected condyle. SPECT (single-photon emission computed tomography) can easily establish the presence of an atypical, prolonged growth exceeding far beyond normal condylar growth and activity. A CT, CBCT, or LDCT (computed tomography, cone-beam computed tomography, or low-dose computed tomography) can confirm the diagnosis by evaluating the scope of bone overgrowth, mandibular basis/ramus asymmetry, tendency to condylar head enlargement, changes in bone density, and occurrence of differences in condylar head shapes, size, and bone structure. In most cases, a condylectomy is the procedure of choice in growing cases of UCH to remove the pathological condyle and reduce asymmetry levels. Sometimes, the growth is very slow and progressive over time, causing slowly growing asymmetry with similar symptoms to any other mandibular asymmetry, and this causes some troublesome procedures in UCH diagnostics, resulting in patients being underdiagnosed; it can even lead to some relapses in mandibular asymmetry and skeletal malocclusion after previously performed orthodontic and surgical treatment of such discrepancies. When the source of asymmetry is not identified in time, possible inadequate treatment protocols can be used. If any relapse of facial and mandibular asymmetry re-occur, SPECT and CT evaluation are necessary to evaluate if condylar hyperplasia is present and to establish what kind of surgical intervention should be used in each case.

**Figure 1:**
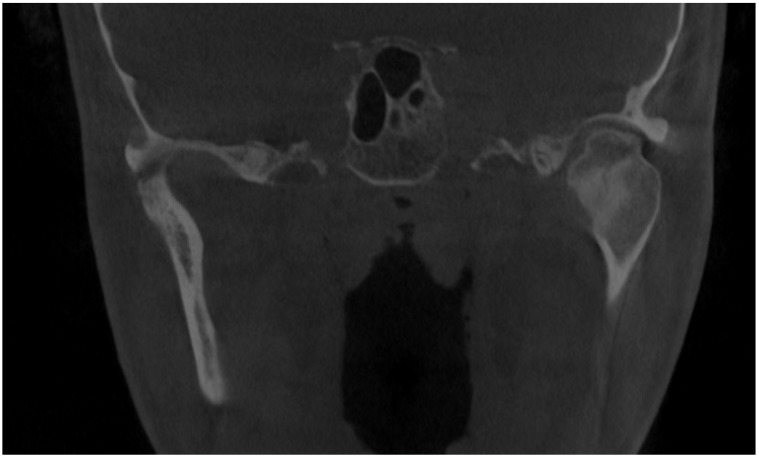
Initial scan—CBCT, coronal view—enlargement and overgrowth of left condylar head causing visible mandibular and skeletal asymmetry. The patient underwent orthodontic treatment (2006–2010) and scheduled BSSO (bilateral sagittal split osteotomy) for surgical correction of the asymmetrical mandibular and featured dentofacial deformity. Six years after the procedure, the patient demonstrated signs of re-occurrence of mandibular asymmetry. After some corrective orthodontic approaches, consultations, and treatment proposals, the patient was scheduled for consultation in our ward (2021–2022). Because of severe mandibular asymmetry, unilateral open bite, chin deviation, midline shift towards the healthy right side, enlargement and overgrowth of the left mandibular basis and ramus, a suspicion of condylar hyperplasia was raised [1,2]. Because the CBCT scans revealed overall extensive progressive growth of the left condylar head, a decision for additional SPECT was decided. According to the known literature, a one-sided open bite with chin deviation towards the heathy opposite side, followed by mandibular corpus enlargement and elongation, are quite common clinical syndromes of condylar hyperplasia. The scope of visible changes in mandibular anatomy is greatly dependent on the time of this abnormal pathological growth and its intensity. It is quite important to evaluate each patient individually because mandibular abnormal growth might not only lead to dentoalveolar changes but also skeletal changes, which, depending on their intensity, might require some degree of surgical intervention. Rarely, condylar hyperplasia might present itself as an osteochondroma or other rare temporomandibular joint tumors [1,2,3]. Therefore, each case of asymmetry, bone change, visible bone enlargement, and overgrowth should be carefully evaluated. Abbreviations: CBCT—cone beam computed tomography, SPECT—single-photon emission computed tomography.

**Figure 2:**
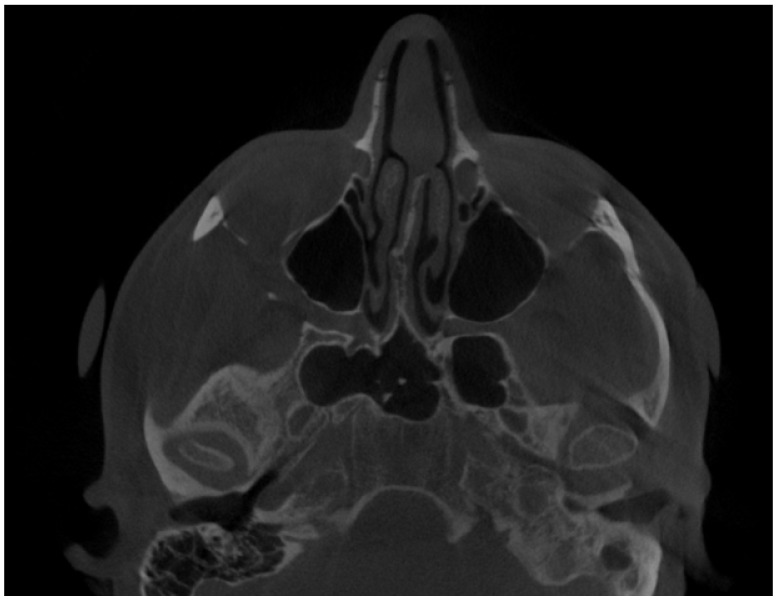
CBCT axial view—enlargement of the left mandibular condylar head. Atypical osteophytes or bone irregularities can have different shapes and sizes while the condylar head is evaluated. The condylar head might have a different shape, size, and contour compared to the opposite healthy side.

**Figure 3:**
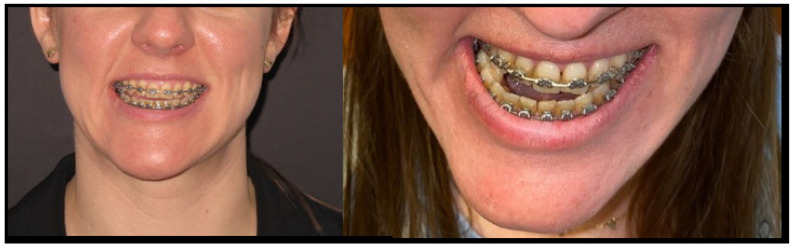
Patient with visible mandibular and facial asymmetry. The mandibular oval and proper facial contour are disrupted by the right mandibular shift because of an increased inappropriate growth occurrence in the affected left mandibular condylar head. The chin is deviated towards the right healthy side, while the left mandibular body is rotated and slightly overgrown. The skeleta, teeth, and soft tissue midline are shifted towards the right side. The open bite is present at the anterior and left part of the mandible; however, some other bite features are corrected due to the use of orthodontic treatment, which prepares the patient for orthognathic surgery. In cases of maxillary asymmetry and deviated maxillary bite plane, the patient should also be scheduled for maxillary Lefort I osteotomy, not only the BSSO—bilateral mandibular sagittal split osteotomy. In cases of severe overgrowth and asymmetries, adjunctive procedures such as genioplasty, mandibular basal marginectomy, or even chin-wing osteotomies are also necessary to improve the facial oval and contour. In most cases, condylectomy is the first surgery, and after at least 4–6 months, a second procedure is scheduled. Few authors advise all-in-one surgery; however, joint instability after a condylectomy might require some additional intermaxillary fixation and maintaining of good jaw stability and bite outcomes after a full osteotomy protocol. Each case should be individually evaluated and planned.

**Figure 4:**
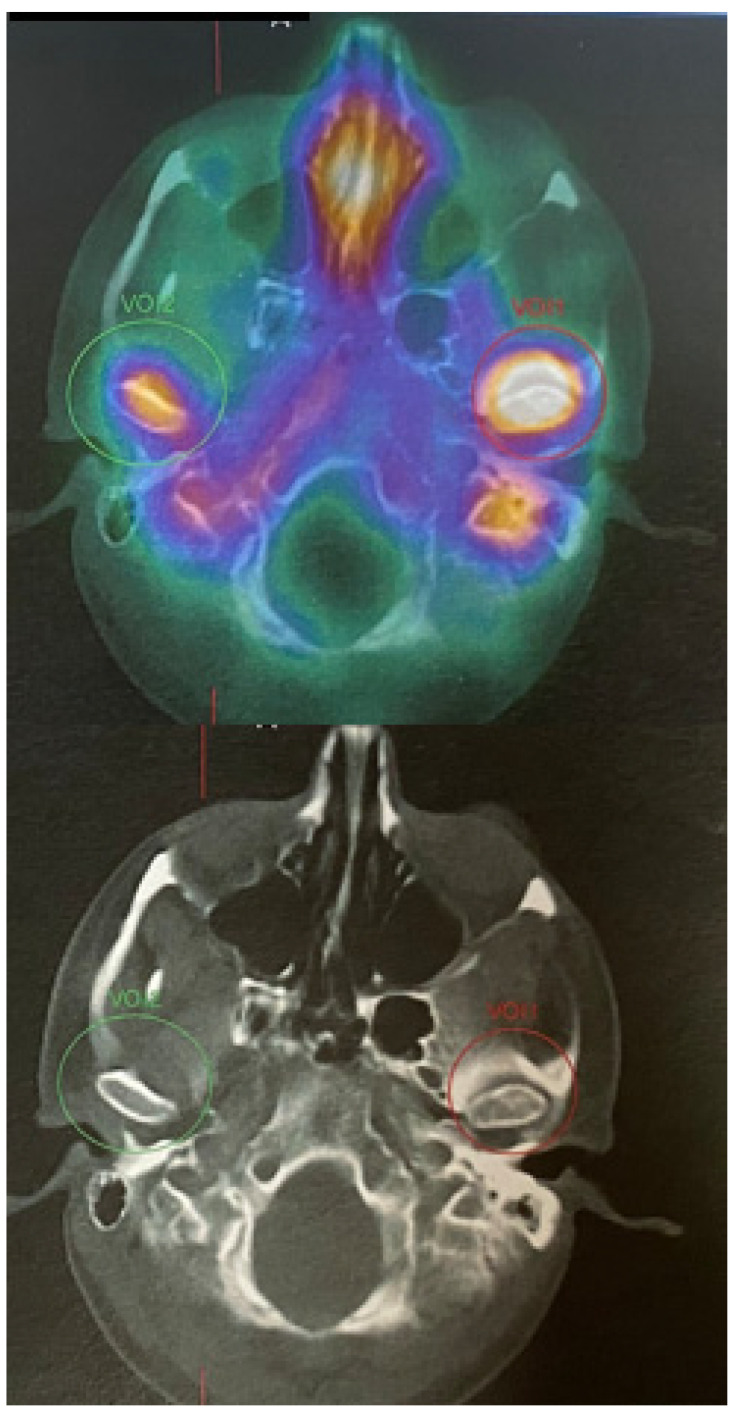
SPECT-CT of the facial skeleton revealed an atypical, extensive, and progressive accumulation of growth in the left mandibular condyle head. The MDP Tc99m (Technetium 99m methylene diphosphonate) is quite important because any accumulation within the affected condyle might either mean inflammation, bone growth, abnormal bone growth, bone tumor, joint overload, some atypical tumors/bone metaplasia, or other findings when the craniofacial skeleton is evaluated. Some authors suggest that the differences in radio uptake of more than 10–15% might suggest growing condylar hyperplasia; however, a full clinical, radiological and SPECT comparison should be drawn at least two times in 4–6 months’ time to compare their features. SPECT-CT alone is not enough to confirm any UCH pathology; therefore, a detailed CT/CBCT/LDCT evaluation and clinical patient examination can greatly influence the future final identification of the disease. Many authors emphasize that SPECT is a very important diagnostic tool and should be always performed in cases of any skeletal asymmetries, especially mandibular asymmetry [4,5]. Abbreviations: UCH—unilateral condylar hyperplasia; CT/LDCT—computed tomography/low-dose computed tomography; green circle–negative growth; red circle–proggresive bone growth in SPECT.

**Figure 5:**
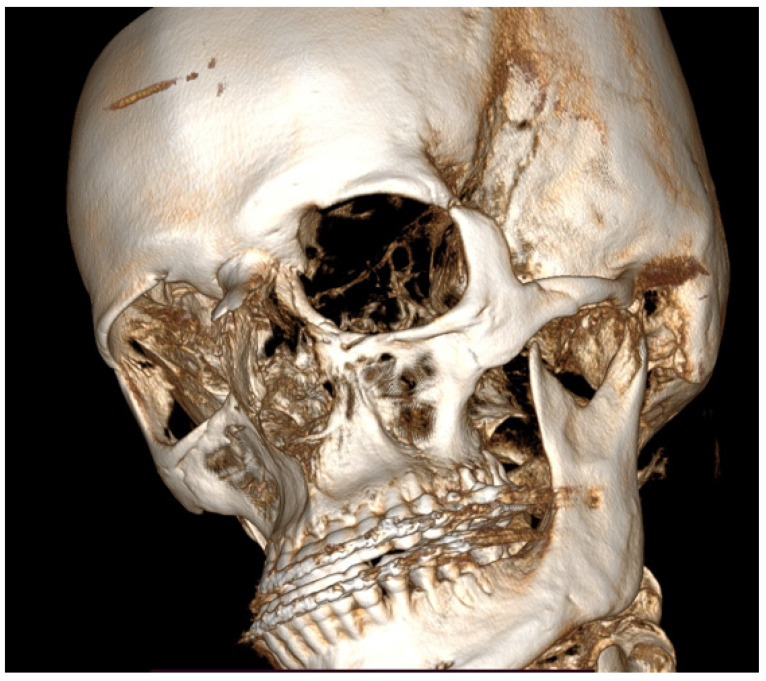
A CBCT-3D reconstruction with visible enlargement and overgrowth of the left mandibular condyle head. Also, a tendency for skeletal class III deformity is visible. In such cases of severe skeletal asymmetries, a necessity for orthognathic surgery procedures is quite important to improve facial symmetry, oval, and contour. The presented images are quite interesting because the unilateral condylar hyperplasia in this case slowly grew over the years and was not found before the first orthognathic procedure in the past; this was the main source of skeletal asymmetry relapse over the next years.

**Figure 6:**
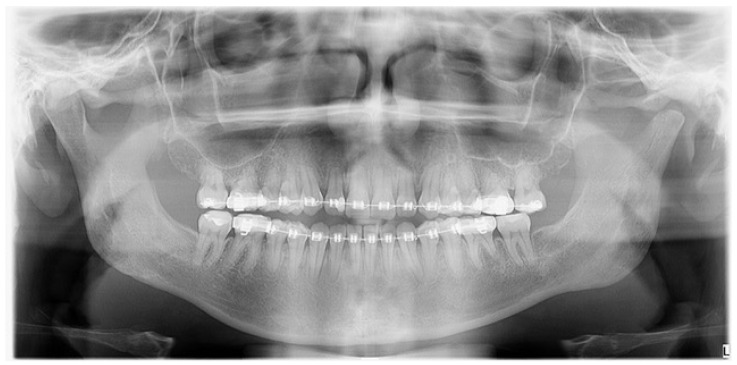
A control panoramic radiograph after six months after a left-sided condylectomy. The jawbone remains asymmetrical with a deviated midline and inappropriate bite; therefore, the patient was scheduled for a revision BSSO surgery to establish more accurate jaw proportions. The scope and degree of the excised affected condylar head influences the later shape and size of the newly formed condylar head stump. Most authors recommend its 5 mm bone removal; however, in this presented case and in others, surgery excision must be bigger and is case-related. Achieving a stable position of the condylar head inside the glenoid fossa is an important matter [3,4,5].

**Figure 7:**
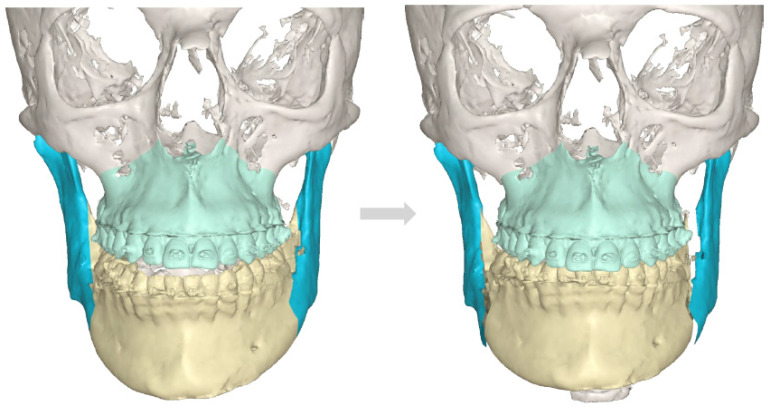
The final outcome for each asymmetry treatment should be focused on restoring facial balance, oval, symmetry, and improved bite. In the presented figure, both the anterior open bite and mandibular shift towards the right healthy side were successfully achieved in one surgery, meeting the patient’s expectations. Since UCH cases are not that common and can be easily misdiagnosed as normal bone asymmetries, laterognathia, or similar cases, it is quite important to plan each surgery step-by-step in CT/CBCT and compare the 3D dimensions of each mandibular body, ramus, and condyle. Each case of atypical asymmetry should be scheduled for a SCPECT-CT evaluation and a condylectomy procedure when the growth is present (min > 10%), active during a period of time, and not decreasing in time. Some authors reported that UCH can be found in patients between 12 and 50 years of age, depending on the scope of pathological abnormal growth and its occurrence in time (rapid growth, slow progression in time, growth cessation) [1,2,3,4,5]. Sometimes, a condylectomy is sufficient enough to restore mandibular balance when skeletal disproportions are not large enough. If the dento-alveolar discrepancies are severe, then the classic osteotomy protocol of orthognathic surgery should be used after patient orthodontic preparation (consisting of Lefort I maxillary osteotomy, BSSO of the mandible, and chin genioplasty). Rarely, when a balanced profile and proper bone proportions are not met, a third surgery focused on facial contouring and re-balancing is necessary (marginectomies, ostectomies, chin wing or others, bone grafting, or implants). In most cases, each clinician should deal with each patient individually because the scope of growth, bone changes, present skeletal malocclusion, bone over-growths, and related changes to both the skeletal profile and even maxillary cant deviation might necessitate a different protocol in each case.

## Data Availability

The datasets used and/or analyzed during this current study are available from the corresponding author upon reasonable request.
